# Prognostic Value of the Systemic Inflammation Response Index in Patients with Adenocarcinoma of the Oesophagogastric Junction: A Propensity Score-Matched Analysis

**DOI:** 10.1155/2019/4659048

**Published:** 2019-11-04

**Authors:** Yuan Chen, Ming Jin, Yingjie Shao, Guoping Xu

**Affiliations:** ^1^Department of Radiation Oncology, The Third Affiliated Hospital of Soochow University, 185 Juqian Road, Changzhou 213003, China; ^2^Department of Clinical Laboratory, The Third Affiliated Hospital of Soochow University, 185 Juqian Road, Changzhou 213003, China

## Abstract

Systemic inflammation is closely related to the occurrence and development of tumours. Based on preoperative neutrophil, monocyte, and lymphocyte counts, a new systemic inflammation response index (SIRI) was established, and the predictive ability of the SIRI for the survival of patients with adenocarcinoma of the oesophagogastric junction (AEG) was evaluated by propensity score matching (PSM) analysis. A total of 302 AEG patients undergoing radical surgery were studied. Univariate and multivariate analyses were performed using Cox proportional hazards regression models. Time-dependent receiver operating characteristic (ROC) curves were used to compare the predictive capabilities of the SIRI. PSM was implemented to balance the baseline characteristics. The results showed that the SIRI, PLR, NLR, and MLR were associated with overall survival (OS) in AEG patients based on the Kaplan-Meier survival analysis. Multivariate analysis demonstrated that the SIRI was an independent prognostic factor. The AUC for the SIRI was significantly greater than that for the NLR, PLR, and MLR in predicting the 3- and 5-year OS of AEG patients. In PSM analysis, the SIRI remained an independent prognostic indicator of OS in AEG patients. The SIRI is a novel, simple, and inexpensive prognostic predictor for AEG. The prognostic value of the SIRI is superior to that of the PLR, NLR, and MLR. The SIRI can be used to distinguish the prognosis of AEG patients with different TNM stages and can be an important supplement to TNM staging.

## 1. Introduction

In recent years, the incidence of adenocarcinoma of the oesophagogastric junction (AEG) has increased significantly [1]. Due to the particularity of this tumour anatomy, there has been a lack of uniform definition and classification for a long time, and AEG has not been treated as an independent disease [1]. Clinical studies of AEG are relatively few, often including gastric cancer or oesophageal cancer. In related studies, the results lack comparability. Therefore, substantial controversy remains in the choice of the best treatment plan for AEG [1]. With the introduction and practice of the Siewert classification theory [2], an increasing number of studies have regarded AEG as an independent disease. Compared with gastric cancer in other parts, cancer of the oesophagogastric junction is more likely to have deep infiltration into the stomach wall, lymph node metastasis, and recurrence after operation, with worse prognosis [3]. Therefore, choosing the right staging and prognosis for AEG has become a new research interest.

Since inflammation has been identified as an important carcinogenic factor, systemic inflammatory factors have been shown to play an important role in the development and prognosis of malignant tumours [4]. The persistent inflammatory response weakens the body's adaptive immune response and indirectly promotes tumourigenesis and metastasis. In the mid-19th century, Virchow discovered the presence of tumour-infiltrating lymphocytes, which may initially be associated with inflammation [5]. With more in-depth research, it was confirmed that chronic inflammation is involved in the occurrence of tumours. Further research has confirmed that chronic inflammation is involved in various stages of tumourigenesis, proliferation, metastasis, aging, and apoptosis. Inflammation is also considered to be the seventh hallmark of malignant tumours [6]. A growing number of studies have shown that systemic inflammatory response markers, including the neutrophil-to-lymphocyte ratio (NLR), platelet-to-lymphocyte ratio (PLR), and monocyte-to-lymphocyte (MLR), play an extremely important role in tumour development and prognosis [7–9]. In recent years, the systemic inflammation response index (SIRI) based on peripheral blood neutrophil, monocyte, and lymphocyte counts has been shown to be a new prognostic indicator for pancreatic cancer [10], liver cancer [11], nasopharyngeal cancer [12], and oesophageal cancer [13]. However, the prognostic value of the SIRI has not been reported in AEG. This study investigated the factors that influence the prognosis of AEG patients and evaluated whether the SIRI is better at determining the prognosis of AEG patients better than the NLR, PLR, and MLR. In addition, to illustrate the statistical capabilities of the SIRI, we performed a propensity score matching (PSM) analysis.

## 2. Patients and Methods

### 2.1. Patients

The clinical data of AEG patients treated with radical surgery at The Third Affiliated Hospital of Suzhou University from January 2008 to December 2010 were retrospectively analysed. The inclusion criteria were as follows: (1) patients undergoing radical surgery, with clear histopathological diagnosis; (2) patients with complete follow-up data and clear end points; and (3) patients with complete preoperative laboratory test results. The exclusion criteria were as follows: (1) patients with a history of infectious disease within 1 month before surgery; (2) patients with a history of previous rheumatoid immune disease; (3) patients with incomplete data; (4) patients undergoing neoadjuvant chemotherapy or preoperative radiotherapy; and (5) patients with survival for more than 3 months after surgery. According to the above inclusion and exclusion criteria, a total of 302 patients were enrolled in the study. Tumour staging was performed according to the American Joint Committee on Cancer (AJCC) TNM staging system. All patients were confirmed to suffer from stage 0-III AEG via histopathology. This study was approved by the Ethics Committee of The Third Affiliated Hospital of Suzhou University in accordance with the guidelines of the Declaration of Helsinki, and informed consent was obtained from all patients.

### 2.2. Preoperative Blood Indexes and Optimal Cut-Off Values

Peripheral blood was drawn from patients within 1 week before surgery. The SYSMEX XS-8001 automatic haematology analyser (SYSMEX, Japan) was used to detect platelets, neutrophils, lymphocytes, and mononuclear cells. NLR = neutrophil count/lymphocyte count, PLR = platelet count/lymphocyte count, MLR = monocyte count/lymphocyte count, SIRI = neutrophil count × monocyte count/lymphocyte count. The cut-off values of the optimal SIRI, NLR, PLR, and MLR in the queue were calculated by ROC curve analysis. The choice of threshold based on the Youden index (sensitivity + specificity − 1) was used to estimate sensitivity and specificity. According to the optimal cut-off values, the parameters were as follows: NLR (NLR ≤ 1.7, NLR > 1.7), PLR (PLR ≤ 96, PLR > 96), MLR (MLR ≤ 0.20, MLR > 0.20), and SIRI (SIRI ≤ 0.68, SIRI > 0.68).

### 2.3. Follow-Up

The patients were followed up via telephone calls and outpatient visits. The relevant tumour detection indicators were evaluated via follow-up every 3-6 months after surgery. CT examinations were performed every 6 months to 1 year, and gastroscopy was performed every 1-3 years. The last follow-up was on December 1, 2016. The overall survival time was calculated from the day of surgery to the day of death or final follow-up. All patients were followed up for 4-98 months, with a median follow-up period of 55 months.

### 2.4. Statistical Methods

SPSS 22.0 software was used for statistical analysis. Categorical variables were compared using the chi-squared test. According to the receiver operating characteristic (ROC) analysis, the optimal cut-off value of the total survival time of AEG patients was determined by the MLR, NLR, and PLR, and the prognosis of patients was predicted by the area under the curve (AUC). Survival curves were plotted by the Kaplan-Meier method and compared by the log-rank test. Univariate Cox regression analysis was performed to identify factors affecting survival. The statistically significant variables from the univariate analysis were further validated by Cox proportional hazards models for multivariate analysis. In addition, PSM analysis was performed due to imbalances in baseline characteristics. PSM was performed using the nearest neighbour matching algorithm, allowing the maximum tolerance difference between the propensity scores to be less than 30% of the propensity score SD. Unless otherwise stated, *P* < 0.05 suggested a statistically significant difference.

## 3. Results

### 3.1. Clinical Characteristics of the Patients

A total of 302 patients were included. There was no patient lost to follow-up. All patients were followed up for 4-98 months, with a median follow-up period of 55 months. There were 244 males (80.8%) and 58 females (19.2%) aged 43-84 years (median age 63 years). In terms of TNM stage, there were 67 stage I cases, 94 stage II cases, and 141 stage III cases. The 3-year and 5-year OS rates were 57.9% and 47.3%, respectively. The relationship between the SIRI, NLR, PLR, and MLR and the clinicopathological features of AEG patients is shown in Tables 1 and 2. As shown in Table 1, the SIRI of AEG patients was related to sex, grade, tumour size, T phase, N phase, TNM phase, and vascular invasion (*P* < 0.05). In addition, relationships among the SIRI, PLR, NLR, and MLR were also studied (Table 2), and it was found that the SIRI was significantly associated with other systemic inflammatory markers (*P* < 0.001).

### 3.2. Value of the SIRI, NLR, PLR, and MLR in the Prognostic Prediction of AEG Patients

**Figure 1:**
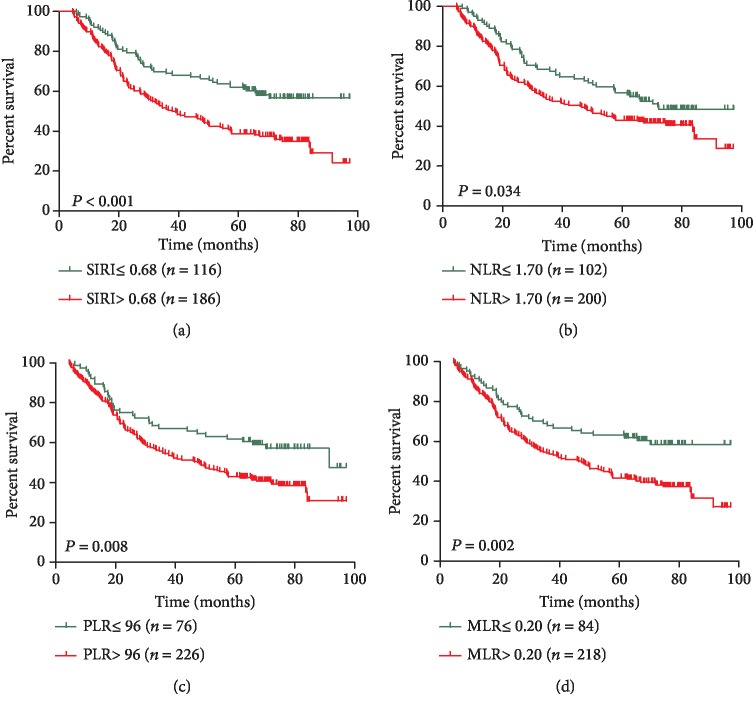
Kaplan-Meier survival curves for patients stratified based on (a) SIRI, (b) NLR, (c) PLR, and (d) MLR in patients with adenocarcinoma of the oesophagogastric junction.

**Figure 2:**
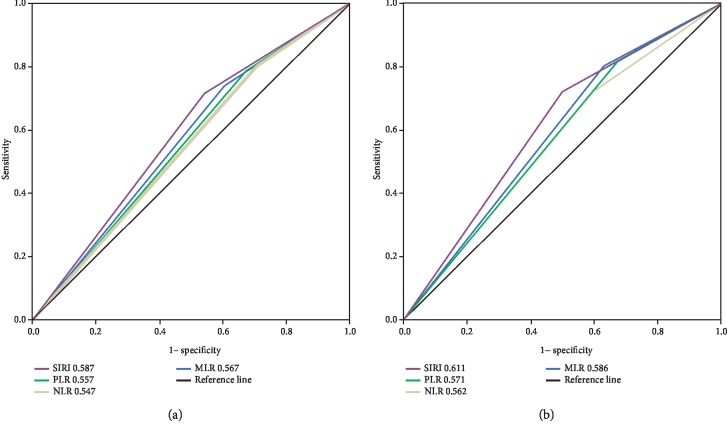
Predictive ability of the SIRI in adenocarcinoma of the oesophagogastric junction was compared with PLR, NLR, and MLR by ROC curves in 3 years (a) and 5 years (b).

**Figure 3:**
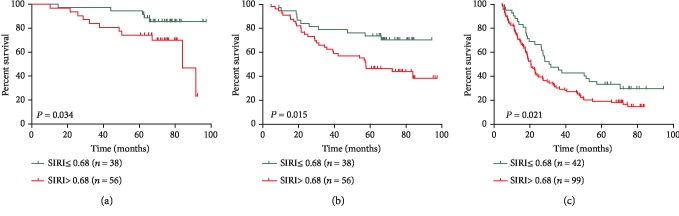
Effect of the SIRI on the survival of adenocarcinoma of the oesophagogastric junction patients in stage I (a), stage II (b), and stage III (c).

Kaplan-Meier survival analyses showed that AEG patients with a higher SIRI (>0.68) had worse OS than AEG patients with a lower SIRI (≤0.68) (*P* < 0.001, Figure 1(a)). High NLR, PLR, and MLR were also correlated with OS (*P* = 0.038, *P* = 0.024, and *P* = 0.008, respectively) to a lesser degree (Figures 1(b)–1(d)). In univariate analysis, we found that grade, tumour size, TNM stage, vascular invasion, SIRI, PLR, NLR, and high calcium levels were important prognostic factors (Table 3). In the multivariate analysis, TNM stage, vascular invasion, and SIRI were identified as independent prognostic factors (Table 3). Among the four inflammation-based prognostic indicators, only the SIRI was an independent risk factor for AEG patients (Table 3). To compare the predictive power of the PLR, NLR, and high calcium levels, we statistically compared the areas under the ROC curves at 3 and 5 years of follow-up. The results showed that at 3 years of follow-up, the AUC of the SIRI was significantly larger than that of the NLR, PLR, or MLR, and the survival rate of AEG patients over 5 years was higher than that of NLR, PLR, and MLR (Figures 2(a) and 2(b)), suggesting that the SIRI has a better prognostic value in predicting 3-year and 5-year survival rates in AEG patients than the NLR, PLR, or MLR. After identifying different subgroups based on TNM stages, we performed survival analysis to determine whether the SIRI could further differentiate patient prognoses. It was observed that in patients with stage I, II, and III diseases, high SIRI values were significantly associated with poor outcomes (Figures 3(a)–3(c)).

### 3.3. PSM Analysis

**Figure 4:**
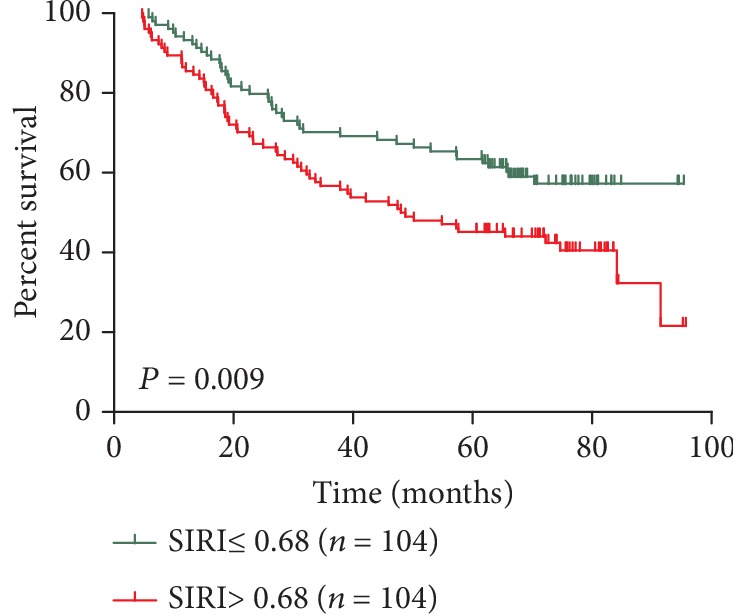
Kaplan-Meier survival curves for patients stratified based on SIRI after propensity matching.

The imbalance between sex, grade, tumour size, T stage, N stage, AJCC stage, and vascular invasion in patients with SIRI ≤ 0.68 and >0.68 may affect the reliability of the results (Table 1). Through PSM analysis, 104 patients with SIRI ≤ 0.68 and 104 patients with SIRI > 0.68 were compared. In the comparative study, the distribution of the main features was basically balanced between the two groups (*P* > 0.3) (Table 1), and survival analysis in the 208 matched patients showed a significant difference between the high SIRI group and the low SIRI group (*P* = 0.009) (Figure 4). In addition, multivariate analysis showed that the SIRI remained an independent predictor of OS in AEG patients (*P* = 0.040) (Table 4).

## 4. Discussion

Throughout the world, the incidence of gastric cancer has decreased year by year, and the incidence of AEG has increased rapidly, and its growth rate has exceeded that of cancer in other parts of the stomach [1]. Due to its unique biological characteristics, the overall prognosis of AEG is worse than that of distal gastric cancer. Siewert et al. reported that in Western countries, more than 80% of AEG is diagnosed at an advanced stage, and the 5-year OS is lower than 30% [14]. Therefore, new treatments and prognostic assessments are urgently needed. In recent years, an increasing number of studies have shown that chronic inflammation is significantly correlated with malignant tumours [4]. Inflammatory cells can alter the microenvironment of the tumour, thereby promoting tumourigenesis and increasing tumour cell proliferation, migration, and immune escape [4]. A number of studies have shown that inflammatory factors can be effectively used to predict the prognosis of cancer patients. Cells currently used to assess systemic inflammation are mainly neutrophils, monocytes, lymphocytes, and platelets in routine blood samples. Previous studies have confirmed that these inflammatory cells are indicative of the NLR, PLR, MLR, and other indicators and can be used to predict tumour prognosis more than single-cell counts [7–9]. Zhang et al. found that the MLR and NLR may be prognostic factors in patients with nonmetastatic Siewert II/III AEG [15]. Another study also confirmed that the NLR can be used to determine the prognosis of AEG patients [16]. Urabe et al. also confirmed that the NLR, PLR, and MLR are significantly associated with OS in AEG patients [17]. Messager et al. found that the PLR is associated with OS in AEG patients and may be a useful prognostic biomarker [18]. Yuan et al. showed that preoperative NLR elevation is an effective marker of tumour recurrence, independently predicting disease-free survival and poor overall survival after R0 resection in AEG patients [19]. These studies confirm the impact of the NLR, PLR, and MLR on the prognosis of AEG patients. The SIRI used in this study combined three inflammatory cell counts, and we confirmed the prognostic value of the SIRI in AEG patients. We found that the SIRI is an independent prognostic factor in AEG patients and that the SIRI has a higher predictive value for prognosis in AEG patients than the NLR, PLR, and MLR. In addition, to exclude confounding factors, we used PSM analysis and found that the SIRI remains an independent prognostic factor for AEG.

As a predictive tool for cancer patients, the SIRI has the following theoretical foundations. First, lymphocytes play an important role in host antitumour immunity, mediating cytotoxic cell death and inhibiting tumour cell proliferation and metastasis [4]. Absolute lymphocyte counts reflect the ability of the body's immune system to respond. A decrease in the number of lymphocytes indicates that the body lacks an effective immune response against the tumour, thereby promoting tumour progression and metastasis. Lymphocytes also inhibit the proliferation and metastasis of tumour cells by participating in cell death caused by cytotoxicity and inducing the secretion of antitumour cytokines [20]. Second, neutrophils play an important role in all stages of tumour progression: (1) they can produce a variety of ligands and secretory MMPs to induce tumour cell proliferation and invasion [21, 22]; (2) they can release angiogenic factors and promote tumour angiogenesis [23]; and (3) they can interact with T cells, thereby affecting tumour cell proliferation, angiogenesis, and metastasis [24]. In summary, neutrophils promote tumour progression at all stages of the tumour. Third, monocytes play an important role in tumourigenesis and metastasis. Tumour-associated macrophages derived from peripheral blood mononuclear cells can inhibit acquired immune responses, promote tumour growth and tumour angiogenesis, and cause tumour invasion and migration [25]. The number of peripheral blood mononuclear cells can reflect the presence and status of tumour-associated macrophages in patients [25]. Therefore, the number of monocytes in the peripheral blood of a patient with tumour can reflect the patient's potential inflammatory state. The SIRI indicators used in this study systematically reflect the complex interaction and potential synergy among neutrophils, monocytes, and lymphocytes in the tumour microenvironment, reflecting the balance between the host inflammatory response and immune response status. Target markers and the SIRI are readily available in an almost noninvasive, low cost, and reproducible manner. Therefore, it is believed that the SIRI is a good indicator for predicting the prognosis of AEG patients.

Although the results are satisfactory, there are still some limitations. First, this is a single-centre retrospective study with possible selection biases, which may be confused with detection bias and analytical bias, and thus, the reliability of the conclusions is much lower than that of randomized controlled trials. Second, the cut-off values based on the prognostic inflammatory indicators reported in the literature are different. Third, some studies have found the prognostic value of the ratio CRP/Albumin in tumours, such as oesophageal squamous cell cancer [9, 26], ovarian cancer [27], and colorectal cancer [28]. We have tried to add CRP/Albumin analysis in our study. But we did not have enough data for analysis. We suggested further research on the prognostic value of CRP/Albumin in AJE. Finally, there is some heterogeneity in the treatment received by patients after surgical resection, which may lead to different clinical outcomes. In summary, we hope a large multicentre randomized comparison can be made to verify our results in the future.

## 5. Conclusion

Systemic inflammatory responses in AEG patients can reflect postoperative survival; in particular, a high SIRI is an independent prognostic factor for AEG patients. In addition, the SIRI can be used to distinguish the prognosis of patients with different TNM stages and can be an important supplement to TNM staging. In conclusion, the SIRI is a noninvasive, accessible, low-cost, and universally available method with broad application prospects in AEG patients.

## Figures and Tables

**Table 1:** Baseline characteristics for patients with SIRI ≤ 0.68 versus SIRI > 0.68 before and after propensity matching.

Clinical parameter	Unmatched (complete) dataset				Matched (1 : 1) dataset					
	SIRI ≤ 0.68 (116)	SIRI > 0.68 (186)	*χ* ^2^	*P*	SD	SIRI ≤ 0.68 (104)	SIRI > 0.68 (104)	*χ* ^2^	*P*	SD
Sex			14.60	<0.001^∗^				0.12	0.734	
Male	81	163			0.08	81	83			0.09
Female	35	23			0.08	23	21			0.09
Age			1.89	0.169				0.02	0.887	
≤60	51	67				42	41			
>60	65	119				62	63			
Grade			8.33	0.016^∗^				2.06	0.356	
Well	11	4				9	4			
Moderately	77	129				70	73			
Poorly	28	53				25	27			
Tumour size			6.67	0.010^∗^				0.92	0.337	
≤5 cm	90	118			0.70	81	75			0.08
>5 cm	26	68			0.70	23	29			0.08
T stage			9.23	0.026^∗^				3.35	0.340	
Tis-T1	24	19			0.36	22	19			0.07
T2	16	17			0.10	14	11			0.01
T3	47	89			0.08	43	37			0.10
T4	29	61			0.15	25	37			0.15
N stage			10.21	0.017^∗^				3.61	0.306	
N0	58	64			0.28	55	42			0.03
N1	22	42			0.10	18	26			0.12
N2	23	37			0.08	18	20			0.02
N3	13	43			0.01	13	16			0.16
AJCC stage			11.24	0.004^∗^				1.06	0.588	
I	36	31			0.29	33	27			0
II	38	56			0.09	35	35			0
III	42	99			0.05	36	42			0
Vascular invasion			7.86	0.005^∗^				0.74	0.390	
No	111	159			0.20	99	96			0.09
Yes	5	27			0.12	5	8			0.11

SD: standard deviation; AJCC: American Joint Committee on Cancer; SIRI: systemic inflammation response index.

**Table 2:** Relationship between NLR, PLR, or MLR and clinicopathological characteristics of patients with adenocarcinoma of oesophagogastric junction.

Clinical parameter	NLR				PLR				MLR			
	≤1.70 (102)	>1.70 (200)	*χ* ^2^	*P*	≤96 (76)	>96 (226)	*χ* ^2^	*P*	≤0.20 (84)	>0.20 (218)	*χ* ^2^	*P*
Sex			14.70	<0.001^∗^			6.21	0.013^∗^			20.44	<0.001^∗^
Male	70	174			54	190			54	190		
Female	32	26			22	36			30	28		
Age			0.05	0.831			2.40	0.122			0.70	0.403
≤60	39	79			24	94			36	82		
>60	63	121			52	132			48	136		
Grade			0.61	0.737			0.66	0.720			5.63	0.060
Well	6	9			5	10			8	7		
Moderately	71	135			52	154			57	149		
Poorly	25	56			19	62			19	62		
Tumour size			3.15	0.076			3.63	0.057			0.35	0.552
≤5 cm	77	131			59	149			60	148		
>5 cm	25	69			17	77			24	70		
T stage			7.00	0.072			6.15	0.104			5.66	0.130
Tis-T1	20	23			16	27			18	25		
T2	15	18			11	22			9	24		
T3	39	97			28	108			37	99		
T4	28	62			21	69			20	70		
N stage			2.20	0.533			2.50	0.476			5.62	0.132
N0	46	76			36	86			42	80		
N1	22	42			14	50			18	46		
N2	19	41			15	45			12	48		
N3	15	41			11	45			12	44		
AJCC TNM stage			7.85	0.020^∗^			7.01	0.030^∗^			7.22	0.027^∗^
I	32	35			23	44			26	41		
II	30	64			27	67			28	66		
III	40	101			26	115			30	111		
Vascular invasion			1.23	0.267			0.78	0.376			4.18	0.041^∗^
No	94	176			70	200			80	190		
Yes	8	24			6	26			4	28		
SIRI			99.23	<0.001^∗^			29.16	<0.001^∗^			89.02	<0.001^∗^
≤0.68	79	37			49	67			68	48		
>0.68	23	163			27	159			16	170		
PLR			26.42	<0.001^∗^			—	—			22.03	<0.001^∗^
≤96	44	32			—	—			37	39		
>96	58	168			—	—			47	179		
NLR			—	—			26.42	<0.001^∗^			48.43	<0.001^∗^
≤1.70	—	—			44	58			54	48		
>1.70	—	—			32	168			30	170		
MLR			48.43	<0.001^∗^			22.03	<0.001^∗^			—	—
≤0.20	54	30			37	47			—	—		
>0.20	48	170			39	179			—	—		

AJCC: American Joint Committee on Cancer; SIRI: systemic inflammation response index; PLR: platelet lymphocyte ratio; NLR: neutrophil lymphocyte ratio; MLR: monocyte lymphocyte ratio.

**Table 3:** Univariate and multivariate Cox regression analyses for overall survival in patients with adenocarcinoma of oesophagogastric junction (unmatched complete datasets).

Variables	Univariate analysis		Multivariate analysis	
	HR (95% CI)	*P* value	HR (95% CI)	*P* value
Sex				
Female vs. male	0.96 (0.65-1.42)	0.853	—	—
Age				
>60 vs. ≤60	1.00 (0.74-1.36)	0.999	—	—
Grade		0.001^∗^		0.610^a^
Well	Ref.		Ref.	
Moderately	6.00 (1.48-24.29)	0.012^∗^	1.94 (0.44-8.57)	0.383^a^
Poorly	8.95 (2.18-36.77)	0.002^∗^	2.09 (0.46-9.45)	0.339^a^
Tumour size				
>5 cm vs. ≤5 cm	1.62 (1.19-2.21)	0.002^∗^	1.23 (0.90-1.69)	0.199^a^
AJCC TNM stage		<0.001^∗^		<0.001^∗^^a^
I	Ref.		Ref.	
II	2.31 (1.30-4.11)	0.004^∗^	1.79 (0.97-3.28)	0.062
III	6.50 (3.83-11.02)	<0.001^∗^	4.58 (2.58-8.15)	0.034^∗^
Vascular invasion				
Yes vs. no	3.23 (2.16-4.85)	<0.001^∗^	1.93 (1.26-2.95)	0.003^∗^^a^
SIRI				
>0.68 vs. ≤0.68	1.90 (1.36-2.64)	<0.001^∗^	1.55 (1.10-2.17)	0.011^∗^^a^
NLR				
>1.70 vs. ≤1.70	1.42 (1.03-1.98)	0.035^∗^	1.23 (0.89-1.71)	0.218^b^
PLR				
>96 vs. ≤96	1.66 (1.14-2.43)	0.009^∗^	1.25 (0.89-1.67)	0.253^c^
MLR				
>0.20 vs. ≤0.20	1.82 (1.25-2.65)	0.002^∗^	1.43 (0.97-2.09)	0.071^d^

AJCC: American Joint Committee on Cancer; SIRI: systemic inflammation response index; PLR: platelet lymphocyte ratio; NLR: neutrophil lymphocyte ratio; MLR: monocyte lymphocyte ratio; HR: hazard ratio; CI: confidence interval; Ref.: reference. ^a^The variables (grade, tumour size, TNM stage, vascular invasion, and SIRI) were tested in a multivariate analysis. ^b^The variables (grade, tumour size, TNM stage, vascular invasion, and NLR) were tested in a multivariate analysis. ^c^The variables (grade, tumour size, TNM stage, vascular invasion, and PLR) were tested in a multivariate analysis. ^d^The variables (grade, tumour size, TNM stage, vascular invasion, and MLR) were tested in a multivariate analysis.

**Table 4:** Univariate and multivariate Cox regression analyses for overall survival in patients with adenocarcinoma of oesophagogastric junction (matched datasets, 1 : 1).

Variables	Univariate analysis		Multivariate analysis	
	HR (95% CI)	*P* value	HR (95% CI)	*P* value
Sex				
Female vs. male	1.16 (0.72-1.84)	0.540	—	—
Age				
>60 vs. ≤60	1.09 (0.74-1.62)	0.656	—	—
Grade		0.043^∗^		0.491
Well	Ref.		Ref.	
Moderately	4.56 (1.12-18.56)	0.034^∗^	1.71 (0.38-7.67)	0.486
Poorly	5.23 (1.25-21.99)	0.024^∗^	1.35 (0.29-6.36)	0.703
Tumour size				
>5 cm vs. ≤5 cm	1.52 (1.04-1.98)	0.041^∗^	1.01 (0.66-1.54)	0.96
AJCC TNM stage		<0.001^∗^		<0.001^∗^
I	Ref.		Ref.	
II	1.92 (0.99-3.71)	0.053	1.77 (0.88-3.56)	0.107
III	6.01 (3.36-10.74)	<0.001^∗^	4.97 (2.61-9.46)	<0.001^∗^
Vascular invasion				
Yes vs. no	4.50 (2.58-7.88)	<0.001^∗^	2.38 (1.32-4.29)	0.004^∗^
SIRI				
>0.68 vs. ≤0.68	1.67 (1.13-2.47)	0.010^∗^	1.51 (1.02-2.24)	0.040^∗^

AJCC: American Joint Committee on Cancer; SIRI: systemic inflammation response index; HR: hazard ratio; CI: confidence interval; Ref.: reference.

## Data Availability

The data used to support the findings of this study are available from the corresponding author upon request.
